# Unearthing Optimal Symbiotic Rhizobia Partners from the Main Production Area of *Phaseolus vulgaris* in Yunnan

**DOI:** 10.3390/ijms25158511

**Published:** 2024-08-04

**Authors:** Junjie Zhang, Jingqi Wang, Yufeng Feng, Brigitte Brunel, Xuxiao Zong

**Affiliations:** 1 College of Food and Bioengineering, Zhengzhou University of Light Industry, Zhengzhou 450000, China; 2 Collaborative Innovation Center for Food Production and Safety of Henan Province, Zhengzhou 450002, China; 3 Eco&Sols, University Montpellier, CIRAD, INRAE, Institut Agro, IRD, F-34398 Montpellier, France; brigitte.brunel@supagro.fr; 4 Institute of Crop Sciences, Chinese Academy of Agricultural Sciences, Beijing 100081, China

**Keywords:** common bean, *Rhizobium*, genetic diversity, symbiovar, biogeographic distribution, stress tolerance

## Abstract

*Phaseolus vulgaris* is a globally important legume cash crop, which can carry out symbiotic nitrogen fixation with rhizobia. The presence of suitable rhizobia in cultivating soils is crucial for legume cropping, especially in areas beyond the plant-host native range, where soils may lack efficient symbiotic partners. We analyzed the distribution patterns and traits of native rhizobia associated with *P. vulgaris* in soils of Yunnan, where the common bean experienced a recent expansion. A total of 608 rhizobial isolates were tracked from soils of fifteen sampling sites using two local varieties of *P. vulgaris*. The isolates were discriminated into 43 genotypes as defined by IGS PCR-RFLP. Multiple locus sequence analysis based on *recA*, *atpD* and *rpoB* of representative strains placed them into 11 rhizobial species of *Rhizobium* involving *Rhizobium sophorae*, *Rhizobium acidisoli*, *Rhizobium ecuadorense*, *Rhizobium hidalgonense*, *Rhizobium vallis*, *Rhizobium sophoriradicis*, *Rhizobium croatiense*, *Rhizobium anhuiense*, *Rhizobium phaseoli*, *Rhizobium chutanense* and *Rhizobium etli*, and five unknown *Rhizobium* species; *Rhizobium* genosp. I~V. *R. phaseoli* and *R. anhuiense* were the dominant species (28.0% and 28.8%) most widely distributed, followed by *R. croatiense* (14.8%). The other rhizobial species were less numerous or site-specific. Phylogenies of *nodC* and *nif*H markers, were divided into two specific symbiovars, sv. phaseoli regardless of the species affiliation and sv. viciae associated with *R. vallis*. Through symbiotic effect assessment, all the tested strains nodulated both *P. vulgaris* varieties, often resulting with a significant greenness index (91–98%). However, about half of them exhibited better plant biomass performance, at least on one common bean variety, and two isolates (CYAH-6 and BLYH-15) showed a better symbiotic efficiency score. Representative strains revealed diverse abiotic stress tolerance to NaCl, acidity, alkalinity, temperature, drought and glyphosate. One strain efficient on both varieties and exhibiting stress abiotic tolerance (BLYH-15) belonged to *R.* genosp. IV sv. phaseoli, a species first found as a legume symbiont.

## 1. Introduction

The common bean (*Phaseolus vulgaris* L.), known as green bean, is a leguminous plant of the genus *Phaseolus* [1,2]. *P. vulgaris* is one of the frequently consumed vegetables, native to Mexico and Argentina. *P. vulgaris* was first domesticated in the Americas, including Mexico, Colombia, Ecuador and northern Peru, as well as in the Andean center from southern Peru to northern Argentina [3]. *P. vulgaris* prefers warmth and is not tolerant to frost. It was only at the end of the 16th century that China began to introduce its cultivation. When *P. vulgaris* was introduced from the Americas, it was first introduced and domesticated in Guizhou, Yunnan, Sichuan and the surrounding provinces, before spreading to the northeast [4]. According to reports, beans are grown in more than 90 countries worldwide, with a total planting area of 36.5 million km^2^ and a total output of 31.4 million tons. This accounts for 50% of the total output of all edible beans, second only to the legume crop soybean. The planting area of *P. vulgaris* in China is 10 million km^2^, and the total output is 1.3 million tons [5,6]. At present, *P. vulgaris* is widely cultivated in major producing areas such as northeast and north Xinjiang, and southwest China. Shandong Province and Hebei Province in north China, Heilongjiang Province and Jilin Province in northeast China and Yunnan, Guizhou and Sichuan provinces in southwest China have very rich varieties and types of beans. Yunnan is the province with the largest total planting area and output of edible beans in China, serving as the production base for high-quality fresh bean raw materials in the country. *P. vulgaris* has emerged as an important local cash crop because of its easy cultivation and low planting cost, resulting in a relatively extensive planting area. In some dam areas of Chuxiong, Dali, Baoshan and other places within Yunnan Province, the fresh bean industry is thriving. Cultivation of soft pod vine fresh beans has yielded substantial economic benefits, providing a new way for farmers in alpine mountainous areas to alleviate poverty and achieve prosperity. *P. vulgaris* is not only a vegetable, but also a grain or cash crop for foreign exchange export. Therefore, the vigorous development of bean production holds great significance for China’s agriculture.

Nitrogen-fixing bacteria—known as rhizobia—are a group of Gram-negative bacteria characterized by their ability to fix nitrogen in symbiosis with legumes [7,8]. When rhizobia infect leguminous plants, they induce the formation of root nodules, and within root nodules, rhizobia ultimately differentiate in bacteroids that coexist with plant cells. Bacteroids draw nutrients from plants and obtain an environment suitable for nitrogen fixation, while plants obtain nitrogen nutrients through nitrogen fixation by bacteroids, which reduce atmospheric nitrogen to ammonia forms that are assimilable by the plants [9,10,11,12,13]. 

At present, there are several international studies on the diversity of rhizobia associated with *P. vulgaris*, and different rhizobial species forming symbiosis with *P. vulgaris* have been reported. These include *Rhizobium phaseoli* and *Rhizobium tropici* [14], *Rhizobium acidisoli* [15], *Rhizobium croatiense* and *Rhizobium redzepovicii* [16], *Rhizobium freirei* [17], *Rhizobium leguminosarum* [18], *Rhizobium etli* [19], *Rhizobium hidalgonense* [20], *Rhizobium ecuadorense* [21], *Rhizobium giardinii* and *Rhizobium gallicum* [22], *Rhizobium leucaenae* [23], *Rhizobium lusitanum*, *Pararhizobium giardinii* and *Rhizobium pisi* [24], *Rhizobium azibense* [25], *Rhizobium aethiopicum* [26] and *Rhizobium mesoamericanum* [27]. In addition, Chinese sinorhizobia-nodulating *P. vulgaris* have been found in species of *Sinorhizobium meliloti* [28], *Sinorhizobium americanum* [29] and *Sinorhizobium fredii* [30].

There have been several studies on the diversity of rhizobia associated with *P. vulgaris* in China, revealing various rhizobia populations and distribution. For instance, *Rhizobium vallis* strains were isolated from Yunnan leguminous plants in China [31] while *Rhizobium chutanense* strains were isolated from *P. vulgaris* in Jiangxi Province [32]. In addition, *P. vulgaris*-nodulating rhizobia resources from Heilongjiang, Liaoning and Jiangxi provinces in China were collected, and most of the isolated strains belonged to the genus *Rhizobium.* Among these, the species *R. leguminosarum*, *Rhizobium laguerreae*, *R. phaseoli* and *R. vallis* were identified, along with members of the genus *Bradyrhizobium* [33]. Furthermore, the genera *Sinorhizobium*, *Rhizobium*, *Bradyrhizobium*, *Ochrobactrum* and *Agrobacterium* were isolated from *P. vulgaris* in Shaanxi Province. These included species such as *R. phaseoli*, *R. vallis*, *R. giardinii*, *Rhizobium yanglingense*, *R. leguminosarum*, *Sinorhizobium adhaerens*, *S. fredii*, *Sinorhizobium kunmerowiae*, *Bradyrhizobium liaoningense*, *Ochrobactrum anthropic* and *Agrobacterium radiobacter*, totaling 11 species. These findings highlight the very rich diversity of rhizobia associated with *P. vulgaris* [34].

Across different other countries and continents, *P. vulgaris* can also coexist with a wide variety of rhizobia belonging to different genotypes, genera (*Rhizobium*, *Sinorhizobium* and *Bradyrhizobium*) and species with dominant strains or taxa [35]. In its center of origin, the dominant species among *P. vulgaris* nodules is *R. etli*. However, in some regions of Latin America, the prevalence of native strains (*R. leguminosarum*, *R. gallicum* and *R. giardinii*) can hinder the effectiveness of inoculation with *R. etli*, which is generally used to promote nodulation and nitrogen fixation. In areas where pulses have been cultivated, legume nodules harbor many species other than *R. etli* [24]. *R. tropici*, characterized by high degree of genetic stability, dominates in acidic soils and regions with high-temperatures [36]. In Africa, *R. phaseoli*, *R. etli* and a new *Rhizobium* taxon had a great advantage in forming symbiotic relationships with *P. vulgaris*. Meanwhile, *R. leguminosarum* sv. phaseoli, isolated from Moroccan soil, was found more tolerant to acidic conditions in culture media or sterile soil [18].

With the sustainable development of agriculture in China, the frequent use of chemical fertilizers and pesticides has led to environmental contamination and declining soil fertility. Nitrogen is one of the most restricted nutrients for plant growth, and nitrogen fertilizers incur significant costs in crop production [37,38]. In agriculture, the symbiotic relationship between leguminous plants and rhizobia is the key to agricultural and environmental sustainability [39,40]. This relationship provides a natural and renewable nitrogen resource for crops, which is economical and environmentally friendly [41,42]. The rhizobia-leguminous symbiosis system, renowned for its strongest nitrogen fixation capacity, plays an important role in promoting the ecological restoration of contaminated land, including areas affected by saline conditions, heavy metals and pesticide pollution [43]. Yu and Liu [44] found that enhancing soybean salt tolerance could lead to improved soybean yield in saline soils, thus highlighting the potential for biological improvement. Han [45] collected germplasm resources of elite rhizobia from broad beans in the Qinghai area, and finally screened out the strains with high drought and salt-alkali tolerance, offering a good application prospect. Chi et al. [46] conducted NaCl and drought tolerance tests on 58 peanut-nodulating rhizobia isolated from different regions of Shandong Province, and found diversity in salt and drought tolerance among rhizobia not only throughout the province but also within a same region. Wu et al. [47] screened out excellent rhizobia from soybean nodules with strong resistance to high temperatures, salt, antibiotic, acidic and alkaline conditions, as well as strong resistance to dyes and chemical drugs. Cheng et al. [48] simulated cultivated land with a history of glyphosate use by spraying different concentrations of glyphosate solution on the soil before sowing alfalfa. The results showed that different concentrations of glyphosate had inhibitory effects on the growth and nitrogen fixation of alfalfa, with the inhibitory effect strengthening with increasing glyphosate concentration. Therefore, improving crop salt tolerance, acidity and alkalinity tolerance, high temperature tolerance, drought tolerance and other traits, as well as the comprehensive development of biological treatment of difficult and constrained soils, are major issues for future agricultural development. In this study, an optimal matching experiment between rhizobia and *P. vulgaris* was carried out to screen out rhizobia with high efficiency in nitrogen fixation and strong abiotic stress tolerance. The aim was to improve the yield of *P. vulgaris*, mitigate environmental contamination caused by chemical fertilizers and pesticides and improve the quality of *P. vulgaris* [49,50].

Considering all the aforementioned aspects, and the fact that common bean-nodulating rhizobia in Yunnan (China) have not been systematically studied, we conducted the present study. The aim of this work was to evaluate the diversity, relative abundance and geographic distribution of native rhizobia that nodulate *P. vulgaris* in Yunnan Province. Traditionally, common beans have been grown largely in Yunnan as an economic crop for fresh vegetables and grains, and the cultivation area has rapidly expanded to meet consumer demand. Thus, the taxonomic status of the isolated strains from root nodules was determined through ribosomal intergenic typing, phylogenetic analyses of housekeeping genes (*recA*, *atpD* and *rpoB*), the 16S rRNA gene and symbiotic marker genes (*nodC* and *nifH*). Additionally, the distribution of rhizobia in relation to soil properties and environmental factors, as well as the potential of representative strains to induce effective symbiosis and tolerate abiotic stress, were investigated. 

## 2. Results

### 2.1. Physicochemical Characteristics of Soils and the Environment

All 15 sites differed in pH, as well as in their levels of organic matter (OM), alkaline hydrolyzable nitrogen (AN), available phosphorus (AP), available potassium and total salts (EC) (Supplementary Table S1). Soil samples ranged from acidic (five sites) to neutral (five sites) to slightly alkaline (five sites). Most of them contained an average OM level (2–4% across 11 sites), while two had a slightly low OM level (1.7–1.9%) and one a high level (>5%). The field soil at DLEY contained the highest contents of OM (62.6 g/kg soil) and AN (388.9 mg/kg soil). BSSD contained the highest content of AP (388.9 mg/kg soil) while soil from site CXMD contained the highest AK (358.3 mg/kg soil). At the opposite end, site BSCN exhibited the lowest proportions of AP and OM in soil. The field soil at CXDY had the highest salinity (EC = 819.3 µS/kg soil) and site DLMD had the lowest (69.1). The sites belong to the subtropical highland climate (Cwb, Kôppen classification) with mild temperatures and dry winters. Precipitations varied from moderate (nine sites with 870–985 mm/year) to high (six sites with 1020–1350 mm/year). The altitude of the sampling sites was relatively high (1033–2967 m).

### 2.2. Rhizobial Collection and IGS PCR-RFLP Analysis

In total, 608 rhizobial isolates were obtained from the 15 locations (varying from nine (CXWD) to fifty-two (CXDM and CXLF) isolates/site): 303 were obtained by host trapping with variety 1 (black seeds) and the remaining 305 isolates by variety 2 (white seeds) (Supplementary Table S2). The 608 isolates were distinguished into 25, 23 and 22 RFLP patterns obtained from the restriction enzymes *HaeIII*, *MspI* and *HhaI*, respectively. By combining all RFLP patterns, the IGS PCR-RFLP typing allowed the classification of all isolates into 43 IGS types (Table 1). Among the 43 IGS types, IGS type 1 represented the most abundant population (with 153 isolates), followed by IGS type 2 (63 isolates), type 3 (44 isolates), type 4 (38 isolates), type 5 (26 isolates) and type 6 (19 isolates), while the 37 remaining IGS types represented 265 isolates (Supplementary Table S2). Isolates of IGS type 1 were distributed over twelve sampling sites, with the CXLF and DLMD sites having the highest number (68 isolates, 11.2%). IGS type 2 isolates were detected at sites CXMD (nineteen isolates, 3.1%), CXLF (two isolates, 0.3%), CXDH (nine isolates, 1.5%), CXSB (thirteen isolates, 2.1%), CXNH (four isolates, 0.7%), DLXY (nine isolates, 1.5%), BSLY (four isolates, 0.7%) and BSSD (three isolates, 0.5%) (Supplementary Table S2). Sites CXLF, DLXY and CXNH were inhabited by the most diverse *P. vulgaris*-nodulating rhizobial community with thirteen to seventeen IGS types, while sites CXWD and BSCN both harbored only three to four IGS types, indicating that the richness and evenness of rhizobial IGS types varied across the sampling area (Supplementary Table S2).

### 2.3. Identification of Species by Phylogenetic Analysis of Core Genes

Nearly full-length 16S rRNA genes were successfully amplified and sequenced for 46 rhizobial isolates representing all 43 IGS types and sites of origin (Table 1). The representative isolates divided into three groups (Groups 1–3) in the phylogenetic tree (Supplementary Figure S1). First of all, fifteen representative isolates clustered together with several defined *Rhizobium* species in Group 1 that showed 99.7–100% similarity in their 16S rRNA gene sequences. This clade comprises the type strains of *Rhizobium acidisoli* FH13^T^, *R. anhuiense* CCBAU 23252^T^, *Rhizobium hidalgonense* FH14^T^ and *Rhizobium sophorae* CCBAU 03386^T^. Secondly, five representative isolates clustered in Group 2, which also included the type strains of *Rhizobium dioscoreae* S-93^T^, and *Rhizobium vallis* CCBAU 65647^T^. They shared 98.1–100% similarities with each other. Finally, Group 3 contained 26 representative isolates which shared 99.6–100% similarities with *Rhizobium phaseoli* ATCC 14482^T^, *Rhizobium ecuadorense* CNPSo 671^T^, *Rhizobium chutanense* C5^T^, *Rhizobium bangladeshense* BLR175^T^, *Rhizobium aethiopicum* HBR26^T^, *Rhizobium etli* CFN 42^T^, *Rhizobium sophoriradicis* CCBAU 03470^T^ and *Rhizobium croatiense* 13T^T^. Thus, all the representative isolates had been identified as belonging to *Rhizobium*.

**Figure 1:**
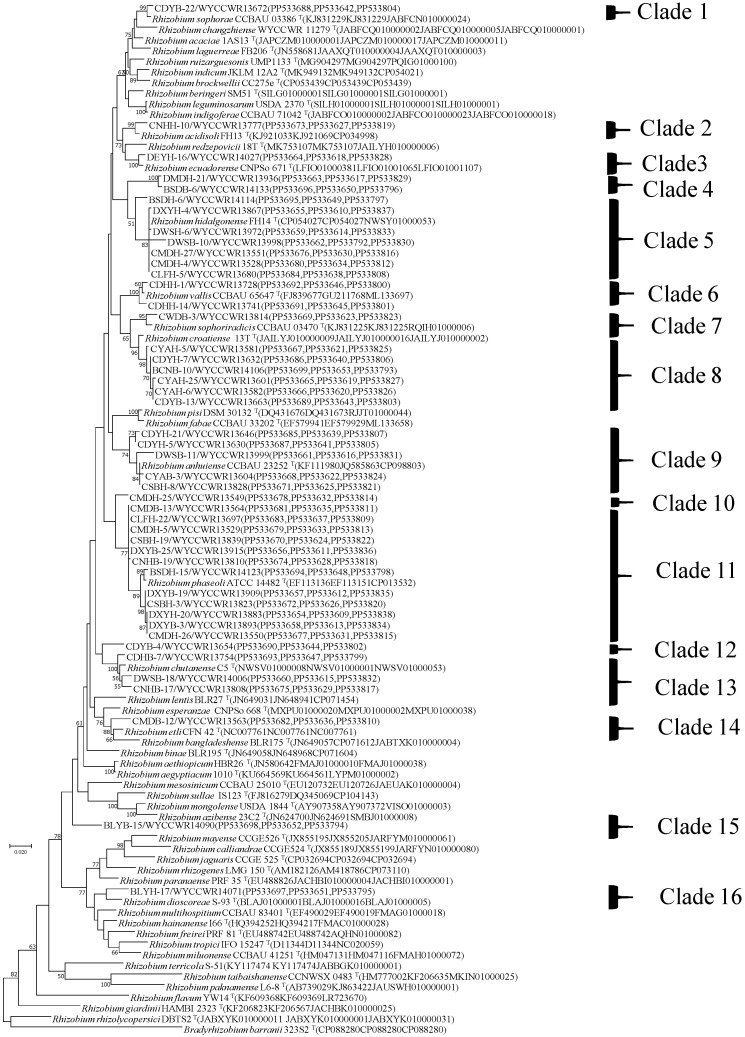
Maximum likelihood phylogenetic tree based on concatenated *recA*-*atpD*-*rpoB* gene sequences (1185 base pairs) showing the relationships of rhizobia isolated from *Phaseolus vulgaris* L. in Yunnan Province of China. The tree was constructed under the best-fit model (GTR + G + I). Scale bar indicates 0.02 nt substitution per site. Bootstrap confidence values (%) calculated for 500 replications > 50% are indicated at the internodes.

The representative isolates were divided into thirteen clades (C1–C16) in the phylogenetic tree based on their concatenated *recA-atpD-rpoB* sequences (Figure 1). C1 includes nine isolates from IGS type 20 with the type strain of *R. sophorae*, sharing 99.1% similarities (Table 1). Thus, cluster C1 was identified as *R. sophorae*. Cluster C2, identified as *R. acidisoli*, contained *R. acidisoli* FH13^T^ and IGS type 18 representing nine isolates with 98.6% similarities. C3, identified as *R. ecuadorense*, contained 11 isolates of IGS type 13, showing 99% similarities with *R. ecuadorense* CNPSo 671^T^. C5 was identified as *R. hidalgonense* and comprised the type strain of *R. hidalgonense* with IGS types 8, 25, 32, 37, 41, 42 and 43 (representing together 32 isolates (5.3% in total) sharing 97.7–100% similarities with each other). Cluster C6, identified as *R. vallis*, contained 14 isolates from IGS types 12 and 39, showing 98.9–99.3% similarities. C7 comprised only the IGS type 28, covering five isolates, and was identified as belonging to the species *R. sophoriradicis* with 98.5% similarity to type strain CCBAU 03470 ^T^. C8, identified as *R. croatiense*, contained *R. croatiense* 13T^T^ and IGS types 3, 6, 15, 21 and 23, totaling 90 isolates (14.8%) with 98.4–98.6% similarities. C9 included four representative isolates from IGS types 1, 11, 29 and 34 (total of 175 isolates, 28.8% in total) identified as *R. anhuiense* with 98.2–100% similarities. C11, identified as *R. phaseoli*, contained *R. phaseoli* ATCC 14482^T^ and IGS types 2, 4, 7, 9, 16, 22, 26, 33, 36, 38 and 40, representing 170 isolates (28.0% in total) with 98.2–99.5% similarities. C13 included three representative isolates from IGS types 5, 27 and 35, identified as *R. chutanense* with 98.6% similarities. C14, identified as *R. etli*, contained four isolates from IGS type 31, showing 97.1% similarities with *R. etli* CFN 42 ^T^. In addition, C4, 10, 12, 15 and 16 (Clade 4, 10, 12, 15, 16) contained six representative strains, and the similarity with the sequences of all known population strains was less than 97%, the model strain with the highest similarity in C4, C12 and C15 was *R. chutanense*, and the similarity was only 94.6–96.3%, and the model bacteria with the highest similarity between C10 and C16 was *R. phaseoli*, with a similarity of only 92.9–96.3%, is suspected to be a new population of rhizobia strains of such groups C4, 10, 12, 15 and 16. The phylogenetic analyses of the single gene of *recA*, *atpD* or *rpoB* are shown in Supplementary Figures S2–S4. 

Group 1 isolates in the 16S rRNA gene phylogenetic tree (Figure S1) contained strains belonging to clades 1, 2, 4 7 and 13 (15 strains). Group 2 (five strains) included strains from clades 7 and 13 along with a strain (CDYB-4) from clade 10. Group 3 (26 strains) encompassed strains from clades 3, 6, 11 and 12, as well as clade 10 except for the strain CDYB-4. Apart from CDYB-4, the phylogenetic 16S rRNA gene tree was consistent with the MLSA classification in clades (Figure 1). Specifically, the CDYB-4 sequence exhibited low bootstrap support in clade 10 (<50%) compared to the other C10 sequences (98%), forming a single branch outside of the defined clades. 

**Figure 2:**
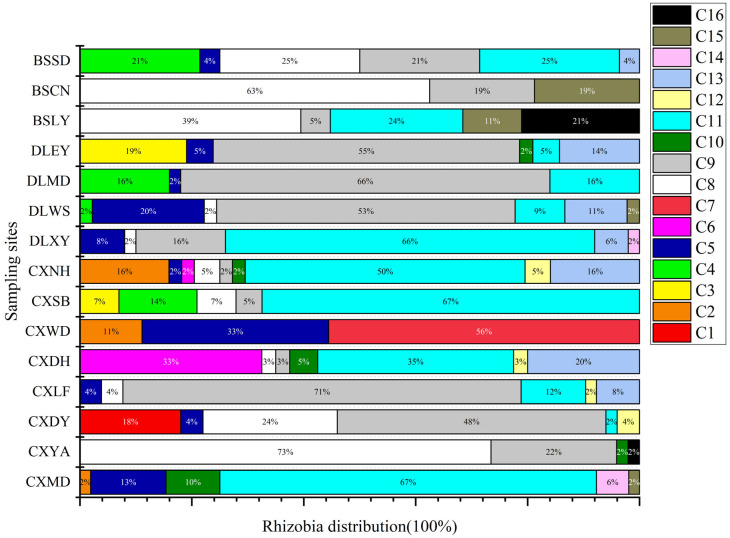
Distribution of core gene clades identifying species of rhizobia isolated from *Phaseolus vulgaris* L. per site and across all sites in Yunnan Province of China. The number of isolates is indicated under the site code. CXSB, Chuxiong Shuang bai, CXWD: Chuxiong Wu ding, CXYA: Chuxiong Yao an, CXMD: Chuxiong Mu ding, CXDH: Chuxiong Dong hua, CXDY: Chuxiong Dayao, CXLF: Chuxiong Lufeng, CXNH: Chuxiong Nanhua, DLEY: Dali Er yuan, DLXY: Dali Xiang yun, DLWS: Dali Wei shan, DLMD: Dali Mi du, BSLY: Baoshan Long yang, BSCN: Baoshan Chang ning, BSSD: Baoshan Shi dian.

Populations of *R. anhuiense* were identified in 13 out of the 15 sampling sites (28.8% in total), with the highest number of isolates, representing the dominant species, observed in CXDY (59%), CXLF (71%), DLWS (53%), DLMD (66%) and DLEY (55%) (Figure 2). *R. phaseoli* was also found dominant (28.0%) and recovered in 12 sampling sites, with populations of CXMD (67%), CXSB (67%), DLXY (66%), CXNH (50%) and CXDH (35%) being the most abundant, establishing it as the dominant species in these five sites. Both *R. hidalgonense* (5.3%) and *R. croatiense* (14.8%) were recovered in 15 sampling sites, with dominance observed at CXYA (73%), CXWD (33%), BSCN (63%) and BSLY (39%) respectively. *R. chutanense* was detected in seven sampling sites, with CXDH (20%) and CXNH (16%) hosting the highest proportion of its members. *R. sophorae*, *R. acidisoli R. ecuadorense*, *R. vallis*, *R. sophoriradicis*, *R. etli* and *Rhizobium* genosp. I~V were found in minority (<2%) in a limited number of sites (one to five). No differences were detected between rhizobia trapped with black beans of variety 1 compared to white beans of variety 2 within clades and sampling sites (Supplementary Figure S5, Table S2), indicating that the population distribution regardless of the common bean variety used for trapping was similar.

### 2.4. Identification of Symbiovars by Phylogenetic Analysis of Symbiotic Genes

Besides the core genes studied above to taxonomically identify rhizobia, it is complementary to type rhizobia according to their symbiovar related to the legume-host spectrum, which does not follow taxonomic phylogeny. To do so, common genes involved in nodulation (*nodC* coding for an N-acetyltransferase) and fixation (*nifH* coding for a subunit of nitrogenase) were investigated in the 46 representative strains. 

**Figure 3:**
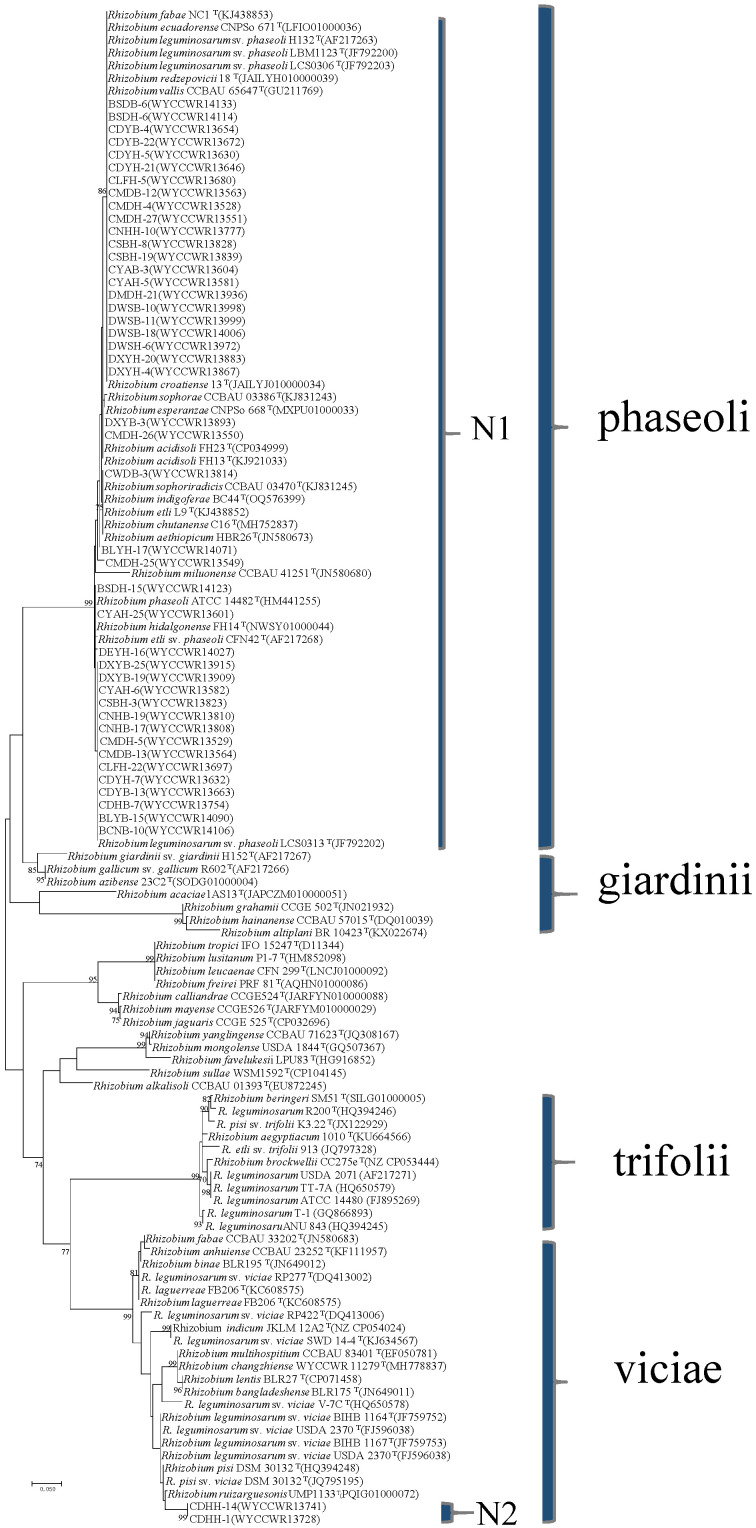
Maximum likelihood phylogenetic tree based on symbiotic gene *nodC* (376 base pairs) showing the relationships of the rhizobia isolated from nodules of *Phaseolus vulgaris* L. in Yunnan Province of China. The two *nodC* groups found among isolates are named N1 and N2. The tree was constructed using the maximum likelihood method under the best-fit model (T92 + G + I). Scale bar indicates 0.05 nt per site. Bootstrap confidence values (%) calculated for 500 replications > 70% are indicated at the internodes.

The *nodC* genes were amplified from all the 46 representative isolates, confirming their genetic basis for rhizobial symbiosis. Phylogenetic analysis defined two strongly supported *nodC* groups (N1-N2) among the representatives (Figure 3). First, the symbiotype N1 (44 representatives) clustered together with *nodC* sequences from strains of several species, all from sv. phaseoli (such as *R. fabae* NC1, *R. croatiense* 13T^T^, *R. sophorae* CCBAU03386, *R. acidisoli* FH13^T^, *R. sophoriradicis* CCBAU 03470^T^, *R. chutanense* C16, *R. phaseoli* ATCC 14482^T^, *R. hidalgonense* FH14^T^, *R. etli* CFN L9^T^ and *R. leguminosarum* bv. phaseoli LCS0313, showing a similarity of 97.5–100%. Second, symbiotype N2 (strains CDHH-1 and CDHH-14) encompassed *nodC* sequences 96% similar to the *nodC* of *R. ruizarguesonis* UMP1133^T^. Thus, all representatives belonged to the sv. phaseoli or sv. viciae (Figure 3). 

Phylogenetic analysis of *nifH* symbiotic gene sequences divided the 46 studied representative strains of *Phaseolus* rhizobia into two distinct groups (Supplementary Figure S6). The larger group clustered 44 representatives with strains *R. chutanense* C5^T^, *R. aethiopicum* HBR26^T^, *R. ecuadorense* CNPSo 671^T^, *R. phaseoli* ATCC 14482^T^, *R. acidisoli* FH23^T^, *R. sophoriradicis* CCBAU 03470^T^ and *R. croatiense* 13T^T^, sharing a sequence similarity of 95.3–100%. The smaller group consisted of two representative strains (CDHH-1 and CDHH-14) associated with *R. lentis* BLR27^T^. These two groups correspond to the *nodC* groups N1 (sv. phaseoli) and N2 (sv. viciae), respectively (Figure 3). 

### 2.5. Correlation Analysis of Rhizobial Distribution with Soil and Environmental Properties

**Figure 4:**
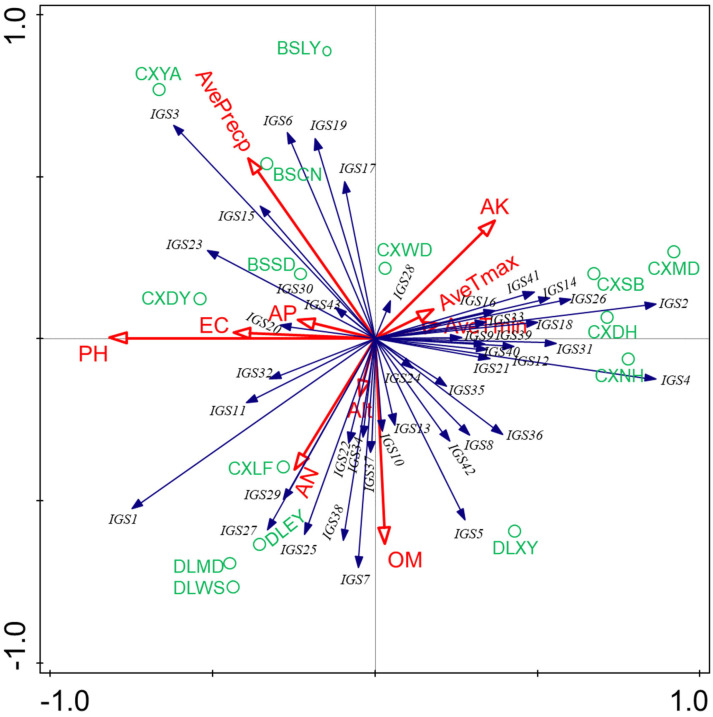
PCA to relate the distribution of the 42 IGS types of isolates (n > 1) to physicochemical factors of soils and environment collected from the different sites. The blue arrows indicate IGS types of rhizobia, green indicate the sampling sites and red arrows represent soil properties and environmental factors. The longer the arrow was, the greater the influence of the soil property and environmental factor presents on the distribution of the IGS types. The smaller the angle between the arrow and the IGS type was, the stronger the effect of the soil property or environmental factor on distribution of the IGS type.

PCA was used to explore the relationships between soil and environment properties, and the rhizobial community composition based on IGS genotypes. The PCA results (Figure 4) showed that the soil chemical factors had different effects on the distribution of the rhizobia populations and IGS types. The IGS types 3, 6, 15, 17, 19, 20, 23, 30 and 43 (left upper part of Figure 4) included 114 isolates recovered mainly in CXDY, CXYA, BSSD, BSCN and BSLY (Supplementary Table S2). Their distribution was positively correlated with pH, EC and average rainfall (AvePrecp), and negatively related with soil OM contents. In particular, IGS20 and IGS15 showed a positive correlation with pH and EC, while IGS3 and IGS20 were associated with the average rainfall (Figure 4). The IGS types 1, 7, 11, 22, 25, 27, 29, 32, 34, 37 and 38 (lower left part of Figure 4) gathered the majority of isolates (222) distributed across four main sites (CXLF, DLEY, DLMD and DLWS) (Supplementary Table S2); they presented a positive association with AN, pH and OM, but were negatively correlated with AK values. In particular, the IGS27 and IGS29 were positively correlated with AN, while IGS37 and IGS7 correlated with higher OM values (Figure 4). The IGS types 5, 8, 10, 13, 24, 35, 36 and 42 (including 82 strains) (lower right part of Figure 4) tended to be associated with DLXY (Supplementary Table S2). Meanwhile, these strains were positively correlated with OM and negatively correlated with EC, pH value and average rainfall. Notably, the IGS10 was positively correlated with OM values (Figure 4). Finally, the IGS types 2, 4, 21, 12, 9, 31, 39, 18, 28, 33, 16, 14, 26, 40 and 41 (middle and upper right part of Figure 4), representing 190 isolates mainly from CXNH, CXDH, CXSB, CXMD and CXWD, were positively distributed with AK, but lower AN, EC and pH values. In particular, IGS28 was positively correlated with AK values. Thus, we found that the different rhizobial populations were influenced differently by edaphic factors, showing potential positive or negative significant correlations. 

*R. sophoriradicis*, *R. acidisoli*, *R. vallis*, *R. etli* and *R.* genosp. II were mainly distributed in CXWD, CXSB, CXDH, CXNH and CXMD. Their distribution was positively correlated with the available potassium content in the soil and negatively correlated with electrical conductivity and pH value. *R. phaseoli*, *R. chutanense*, *R. hidalgonense*, *R. ecuadorense*, *R.* genosp. I and *R.* genosp. III were primarily distributed in the sampling points of DLXY, DLWS, DLMD and DLEY, with a positive correlation with organic matter content and negatively correlated with average precipitation. *R. sophorae* and *R. anhuiense* were mainly distributed in CXDY, CXLF and BSSD, with a positive correlation with alkaline nitrogen, electrical conductivity, and pH value, and a negative correlation with available potassium content. *R. croatiense*, *R.* genosp. IV and *R.* genosp. V were primarily distributed in CXYA, BSCN and BSLY. Their distribution was positively correlated with average precipitation and negatively correlated with the content of organic matter and alkaline nitrogen in the soil. Furthermore, *R. anhuiense* was widely distributed in all 13 sampling points, indicating that these strains were with strong adaptability to different conditions(Supplementary Figure S10).

### 2.6. Symbiotic Efficiency of Rhizobial Representative Strains

All representative isolates from the 42 different IGS types successfully nodulated *P. vulgaris*, with each plant forming more than 50 nodules, and the control group (CK) treated without bacterial treatment did not have nodulation. However, significant variations in symbiotic efficiency, were observed among the nodulated plants (Supplementary Figure S7). The chlorophyll content of *P. vulgaris* plants in the inoculated group was increased (Supplementary Figure S8), showing a significant difference compared to CK, except for strains CDHH-1 and DXYB-19 associated with variety v1 and 4 strains with v2 (CMDH-4, CSBH-8, BSDH-6 and BSDB-6). The average chlorophyll content per black *P. vulgaris* plant of the inoculated strains CDYB-4, CDYH-5, DWSB-18, CDYH-7 and CLFH-5 was among the highest with more than 1.33 times higher than that of CK. Similarly, the chlorophyll content of white *P. vulgaris* inoculated with strains CMDH-27, CYAH-6, BLYH-17, CDYB-13 and DMDH-21 were among the highest. In particular, the average chlorophyll content per plant of CDYB-13 and CNHB-17 was over 1.38 times higher than that of CK. The total mass of black *P. vulgaris* plants inoculated with rhizobia of *P. vulgaris* was significantly improved (Supplementary Figure S9) compared to the control (CK) for 11 strains (CMDH-4, CYAH-6, CDYH-7, CDHB-7, CWDB-3, DXYH-4, DWSB-10, DWSB-11, DWSB-18, DEYH-16 and BLYB-15). Notably, DWSB-18, BLYB-15, CDYH-7, DWSB-11, CMDH-4, DEYH-16, CYAH-6 and DWSB-10 showed dry biomass more than 2.8 times that of CK. Similarly, the total dry weight mass of all white *P. vulgaris* plants inoculated with *P. vulgaris* rhizobia was significantly improved, particularly with strains BLYH-17, CYAH-6, CMDH-25, CDHB-7 and CYAH-25 exceeding 2.5 times that of CK.

**Figure 5:**
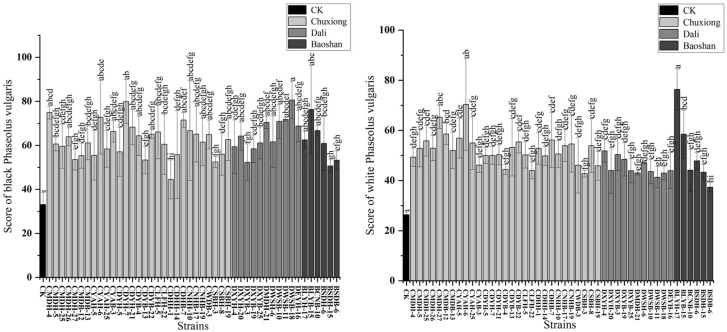
Symbiotic performance score of 46 representative strains based on three symbiotic indexes: chlorophyll index (25%) + number of nodules (25%) + dry plant weight (50%). CK is negative control (uninoculated plants). (**Left**): Scores with black *Phaseolus vulgaris*. (**Right**): Score with white *P. vulgaris*. Treatments (CK or strains) were conducted in triplicates. Bars indicate mean ± standard error. Bars with a same letter are not significantly different (ANOVA + LSD test).

The representative strains were evaluated based on the combined score of the three symbiotic indices mentioned above: chlorophyll (25%) + number of nodules (25%) + total plant dry weight (50%) (Figure 5). Among these, five strains were identified among the high-efficiency nitrogen-fixing group suitable for variety 1 (CMDH-4, CYAH-6, CDYH-7, DWSB-18 and BLYB-15), while another five strains could be designated among the high-efficiency nitrogen-fixing group suitable for variety 2 (CYAH-6, CMDH-27, CMDB-12, BLYH-17 and BLYB-15). Notably, two strains (CYAH-6 and BLYB-15) were identified as high nitrogen-fixing bacteria in both variety 1 and variety 2 (Figure 5).

### 2.7. Abiotic Stress Tolerance of Representative Strains

The experimental results obtained from growth media tests showed that among the 46 strains tested, none could grow at pH 5. Twenty-one strains could grow normally at a pH of 6 (Supplementary Table S3). All strains thrived under pH 7 and pH 8 conditions. Forty strains grew normally at pH 9, while only twenty strains could tolerate pH 10. Remarkably, at pH 11, only nine strains demonstrated normal growth (CMDB-12, CYAH-5, CDYH-7, CDYB-13, CDHH-14, DXYH-4, DXYB-25, BLYH-17 and BLYB-15), indicating their strong alkali tolerance (Supplementary Table S3). All strains exhibited normal grow at a NaCl concentration of 0.01% (control), but as the NaCl concentration increased, strain growth was gradually inhibited. At 1.0% NaCl, only 17 strains could grow normally. When NaCl reached 2.0%, strains CYAH-25 and BLYH-17 showed normal grow and stronger salt tolerance (Supplementary Table S4). None of the strains grew at 4 °C and 10 °C, but all were able to grow at 28 °C. At 37 °C, 12 strains grew normally. At 45 °C, only three strains (CDYH-7, BLYH-17 and BLYB-15) did, indicating their wide temperature adaptation (Supplementary Table S5). All strains were able to grow in presence of 3% and 5% of polyethylene glycol. This number decreased to 31 strains at 7%, and to 11 at 10%. The 11 strains (CMDH-25, CYAH-6, CYAB-3, CDYB-4, CDYB-13, CDYB-22, CDHH-1, CDHH-14, DXYB-25, BLYH-17 and BLYB-15) showed the highest drought tolerance (Supplementary Table S6). The 46 representative rhizobial strains could grow at the lowest concentration of glyphosate but slowly, revealing some growth inhibition in the glyphosate-containing media. This number reached 38 tolerant strains growing in a glyphosate concentration of 1.2 mL/L and 27 strains in 1.8 mL/L, indicating the highest glyphosate tolerance (Supplementary Table S7).

The determination of abiotic stress tolerance across 46 symbiotic strains of *P. vulgaris* showed a rich diversity in terms of adaptability among the different strains. The comprehensive experimental results, encompassing all the abiotic stress tested, i.e., acidity, alkalinity, NaCl, temperature, PEG and glyphosate tolerance, revealed CWDB-3, DXYH-4, BLYH-17 and BLYB-15 as the most tolerant rhizobial strains (Supplementary Table S8). Combined with symbiotic assay results (Section 2.6), BLYB-15 (belonging to *R. dioscoreae*, IGS type 17 according to Section 2.3.) emerged as the strain with both high potential symbiotic efficiency scores on v1 and v2 local bean varieties, together with notable environmental stress tolerance potential (growth at pH 6, pH 11, 45 °C, with 1% NaCl, 10% PEG or 1.8 mL/L glyphosate). 

## 3. Discussion

The study of *P. vulgaris* nodulating rhizobia in Yunnan, combined with the examination of soil physicochemical properties and associated environmental factors, revealed the biogeographic distribution of rhizobia in the area. Unlike previous studies, we systematically investigated the diversity of *P. vulgaris* symbionts at 15 sampling sites in Chuxiong, Dali, and Baoshan areas of Yunnan Province. A total of 608 rhizobial isolates were thus obtained from root nodules and characterized genetically and symbiotically. Taking all the results from the IGS PCR-RFLP typing, phylogenies of 16S rRNA gene sequences, concatenated *recA-atpD-rpoB* sequences, as well as *nodC* and *nifH* sequences, the isolates obtained in this study were classified as a diverse community consisting of 43 IGS types within 16 species all within the genus *Rhizobium*. Most of the representatives selected by IGS type and site belonged to the sv. phaseoli and a minority to sv. viciae. The dominant species among common bean-nodulating rhizobia were *R. anhuiense* (28.8% in relative abundance) and *R. phaseoli* (28.0%), followed by *R. croatiense* (14.8%) and *R. hidalgonense* (5.3%). These species were found to be widely distributed across most of the fifteen sites (11–13 sites). The twelve other species (*R. sophorae*, *R. acidisoli R. ecuadorense*, *R. vallis*, *R. sophoriradicis*, *R. etli*, *R. chutanense* and *Rhizobium* genosp. I~V) were less represented with a narrower distribution (<5.6% and restricted to 1–7 sites). 

The data provide new insights on the diversity structure and geographic distribution of *P. vulgaris*-symbiotic rhizobia in China and areas outside of the plant-host native range. Among the recovered species in Yunnan areas, *R. sophorae* was initially isolated from effective nodules of the shrubby Sophora (*Sophora flavescens*) in Changzhi City (Shanxi Province, China), and was found to be able to effectively nodulate not only Sophora but also *P. vulgaris* [51]. This rhizobial species was later recovered from root nodules of *Vicia faba* L. grown in Panxi (southwest China) [52], southwest China [53], and Hebei Province (northeast China) [54,55]. These findings underscore the widespread existence of *R. sophorae*, emphasizing its adaptability to diverse soil and environmental conditions in China. *R. acidisoli*, initially isolated from *P. vulgaris* nodules in acidic soils of Mexico and forming symbiosis with *P. vulgaris* [15], was later discovered in Morocco [56]. *R. ecuadorense*, isolated from *P. vulgaris* in northern and central Ecuador, had a valuable effective nitrogen fixation with *P. vulgaris* [21]. *R. hidalgonense* was isolated from *P. vulgaris* nodules in the State of Mexico [20]. *R. anhuiense* was first described in rhizobia of pea and *V. faba* in Anhui and Jiangxi Provinces [57], and has been widely recorded in several other provinces in China (provinces of Shandong [58], Sichuan [59] and Hebei [55]). The present study, with a total of 28% relative abundance detected over 13 Yunnan sites, suggests a wide geographic distribution of *R. anhuiense* in China. *R. croatiense* was isolated from *P. vulgaris* landraces from soils of northeast Croatia [16]. *R. vallis*, isolated from nodules of three legume plants (*P. vulgaris*, *Mimosa mimosa* and *Indigofera spicata*) grown in the Yunnan province of China, effectively nodulated *P. vulgaris* but did not nodulate *M. mimosa* and *I. spicata*, suggesting that *R. vallis* could be endophytic in some root nodules [31]. *R. sophoriradicis*, originally isolated from Sophora (*S. flavescens*) [51], has been extensively studied in *P. vulgaris* in Iran [60], South Africa [61] and Peru [62], indicating its widespread distribution among plant species. *R. phaseoli*, which predominates in native areas of common beans, was isolated in Mexico [63], but also from non-native countries such as Ethiopia [26], Brazil [64] and Eswatini [65]. *R. chutanense* was isolated from *P. vulgaris* in Jiangxi Province for the first time, and can effectively trigger nodulation with both *P. vulgaris* and soybean [32]. *R. etli*, rarely found in this study (<1%), was initially discovered in root nodules of the Mexican leguminous plant *Mimosa affinis* and was able to form nodules and fix nitrogen on *M. mimosa* [66]. This species was subsequently found in *P. vulgaris* nodules collected across different agro-ecological zones in Senegal, Gambia (West Africa) [67] and northwestern Argentina. It emerged as a predominant species in common bean nodules from *P. vulgaris* origin areas [24,68] and from different regions of Jordan [69] and Brazil [70]. Moreover, *R. etli* was widely found to be tolerant to high salinity and pH levels in northwestern Morocco [71], Ethiopia [26,72] and Egypt [73,74]. These results show the wide distribution of rhizobia in time and space, as well as its high adaptability to different soil and environmental factors. Adding to the diversity distribution. Notably, *R. tropici* was not observed in this study, similar to findings in the Shaanxi Province of China [34]. However, *R. tropici*, along with *R. etli* and *R. phaseoli*, is another predominant species among *P. vulgaris* symbionts widely recovered in continents and countries such as Columbia [14], Argentina [68], Brazil [64,75] and Iran [60], as well as in north, west or South Africa [61,67,75]. Bacteria of the genus *Rhizobium* have been isolated from a variety of sources and widely utilized for their nitrogen-fixing abilities, both in environment and agriculture. Our findings highlight the broad geographic distribution of these species worldwide, suggesting that *P. vulgaris* plants have selected chromosomal backgrounds or communities suited to local conditions over long-term legume cultivation. Ultimately, the detection of highly conserved *nodC* genes across the 13 rhizobial species identified in this study provides further evidence of *P. vulgaris* plants’ stringent selection of symbiosis genes in their microsymbionts. This selective pressure may favor symbiosis genes toward the most adapted indigenous rhizobia, as reported in other cases [76]. To sum up, our results underscore the necessity of screening and selecting high-quality rhizobial strains with strong adaptability to local conditions for inoculant production aimed at enhancing *P. vulgaris* and other legume yields. 

The values of OM, AN, pH and AK varied significantly among the field soils sampled in this study, and the rhizobial distribution at the IGS type level was found related to these soil characteristics. In addition, we observed that the same species isolated from different soil types had different abundance responses according to soil physicochemical characteristics, as evidenced by a distinct pattern observed at the IGS genotypes within species (Figure 4). Soil chemical and climatic factors exerted varying effects on the distribution of the rhizobial species and IGS types. For instance, we noted a positive correlation between IGS types 3, 6, 15 and 23 (*R. croatiense*), 17 (*R.* genosp. IV), 19 (*R. genosp. V*), 20 (*R. sophorae*), 30 (*R.* genosp. I) and 43 (*R. hidalgonense*) and a negative correlation of IGS types 5, 35 (*R. chutanense*), 24 (*R.* genosp. III), 8, 42, (*R. hidalgonense*), 10 (*R.* genosp. I), 13 (*R. ecuadorense*) and 36 (*R. phaseoli*) with average precipitation (Figure 4). Similarly, a positive correlation was observed between IGS types 2, 4, 9, 16, 26, 33 and 40 (*R. phaseoli*), 12 and 39 (*R. vallis*), 14 (*R.* genosp. II), 41 (*R. hidalgonense*), 18 (*R. acidisoli*), 21 (*R. croatiense*), 31 (*R. etli*) and 28 (*R. sophoriradicis*) and a negative correlation of IGS types 1, 11, 29 and 34 (*R. anhuiense*), 7, 22 and 38 (*R. phaseoli*), 25, 32 and 37 (*R. hidalgonense*) and 27 (*R. chutanense*), with AK, influencing their separation in two distinct distribution patterns. These relationships were consistent with previous reports indicating that soil traits were selective abiotic factors shaping the biogeography of rhizobial species or IGS types [77].

In conclusion, our study demonstrated the existence of indigenous rhizobia that form an effective symbiosis with *P. vulgaris* cultivated in southwest China, in which *R. anhuiense* was newly recorded as *P. vulgaris*-nodulating rhizobia. *R. phaseoli*, *R. anhuiense*, *R. croatiense* and *R. hidalgonense* were the most abundant and widely distributed in the studied soils (28.0%, 28.8%, 14.8% and 5.6%) and formed unique species assemblage of *P. vulgaris*-nodulating rhizobia along with twelve less frequent species (*R. sophorae*, *R. acidisoli*, *R. ecuadorense*, *R. vallis*, *R. sophoriradicis*, *R. etli*, *R. chutanense* and *Rhizobium* genosp. I~V). Moreover *R. anhuiense* occurred in thirteen tested soil types, indicating that strains of these species could be better competitors and adapted to different soil conditions. Finally, all strains tested belonged to the sv. phaseoli regardless of their species affiliation or sv. viciae associated with *R. vallis*. Through symbiotic experiments, the number of nodules per plant was good with chlorophyll index higher than controls for most strains. Dry weights of host plants were significantly improved under inoculation for about half of the *P. vulgaris*-nodulating strains. Therefore, inoculation with rhizobia associated with *P. vulgaris* can significantly promote the growth of *P. vulgaris* plants and achieve effective symbiosis. In parallel, representative strains to IGS types and sites were screened for abiotic stress tolerance, aiming to potentially assist the host legume to cope with soil and environmental stresses. A total of four strains with strong comprehensive tolerance were identified. Combined with the results of symbiotic assay on both *P. vulgaris* varieties, the strain BLYB-15 (*R.* genosp. IV sv. phaseoli, IGS type 17) was found to exhibit high symbiotic efficiency and strong stress tolerance. The resource collection of common bean-associated rhizobia in Yunnan Province, analyzed for genetic and phenotypic diversity, provides significant guidelines for increasing local and sustainable *P. vulgaris* production, with the potential for site-specific selection of efficient rhizobial genetic types.

## 4. Materials and Methods

### 4.1. Field Soil Sampling and Soil and Environmental Characteristics

Soils were sampled from fields cultivated with local common beans (*Phaseolus vulgaris* L.) located in the cities of Chuxiong (CX), Dali (DL) and Baoshan (BS), all in the province of Yunnan, southwest China. A total of 15 sites were studied: 8 at CX (CX-MD, YA, DY LF, DH, WD, SB, NH), 4 at DL (DL-XY, WS, MD, EY) and 3 at BS (BS-LY, CN, SD) (Supplementary Table S1). At each site, soil was collected from the main production area of *P. vulgaris* to a depth of 10–20 cm near the *P. vulgaris* roots during its flowering stage in June 2022. For each site, three randomly taken soil sub-samples of equal volume were crushed to a uniform state, and transported to the laboratory in an ice-filled cooler [77]. Part of each soil sample was chemically analyzed for pH, electrical conductivity (EC), organic matter (OM), available phosphorus (AP), available potassium (AK) and alkaline hydrolyzable nitrogen (AN), as described previously [78]. Using the location information of the sampled sites, annual climate data were collected for each site using DIVA-GIS software Version 7.5 (University of California, Los Angeles, CA, USA). This includes the altitude of the sampling site (Alt), the amount of rainfall throughout the year (AvePrecp), the maximum (AveTmax) and minimum temperatures (AveTmin) and the analysis of climate data [79].

### 4.2. Rhizobial Isolation and Conservation

Surface-sterilized seeds (2.5% *w*/*v* NaClO solution for 5 min) of the common bean (variety 1 of black *P. vulgaris*: garden bean and variety 2 of white *P. vulgaris*: edible pod bean from the local areas of Yunnan Province) were germinated and the seedings were sown in surface-sterilized plastic pots (15 cm high × 10 cm diameter) filled with each sampled soil mixed with sterilized vermiculite (1/5 *v*/*v*). The three randomly taken soil sub-samples were thoroughly mixed to constitute a representative soil sample per field site. For each representative soil sample, 10 repetitions of tracking experiments were performed. All plants were grown under greenhouse conditions of 25/20 °C (day/night) with a 16 h photoperiod. Sterilized water was added to the pots throughout the experiment as required. After 45 days, all plants in all soils were uprooted and bacterial strains were isolated from nodules according to the standard protocol [53]. Five plants from each sampled soil were selected for excision of root nodules and isolation of rhizobia. Root nodules were surface-sterilized, then each individual sterilized root nodule was crushed in sterile water and the bacterial suspension was streaked onto a Yeast extract Mannitol Agar (YMA) plate. After incubation at 28 °C for 2 to 3 days, single colonies representing the dominant bacteria on each plate were picked up and purified by cross-streaking on new YMA plates until pure cultures were visually obtained. All purified isolates were conserved in Tryptone Yeast (TY) broth (tryptone 5 g; yeast extract 3 g; CaCl_2_ 0.6 g; distilled water 1 L, pH 7.0) supplied with glycerol (20%, *v*/*v*) and stored at −80 °C for long-term storage. Additionally, they were maintained on YMA slants at 4 °C for temporary storage.

### 4.3. Genomic Characterization of Rhizobial Isolates

The genomic DNA of each isolate was purified according to Terefework et al. [80,81]. DNA was used as template for PCR amplifications of the 16S-23S rRNA intergenic spacer (IGS) region with primers IGS1490 (forward, TGCGGCTGGATCACCTCCTT) and IGS132′ (reverse, CCGGGTTTCCCCATTCGG) [82]. PCR amplification was carried out in a standard 50 μL reaction mixture, including 1 μL of DNA template and 5 U of *Taq* DNA polymerase (Sangon Biotech (Shanghai) Co., Ltd., Shanghai, China). Aliquots of amplified PCR products (900 base pairs) were visualized after electrophoresis in a 1.0% (*w*/*v*) agarose gel labeled with GoldView type I. Then, PCR products were digested separately with the endonucleases *HaeIII*, *MspI* and *HhaI* [83] at 37 °C for 10 h. The 16S-23S rRNA gene IGS type of each strain was designated after separation and visualization of restriction fragments by electrophoresis in 2.5% (*w*/*v*) agarose gel and UV-illumination.

### 4.4. Molecular and Phylogenetic Identification of the Isolates, Alpha-Diversity Estimation

Isolates sharing the same RFLP pattern of 16S-23S rRNA gene IGS in this study were designed as an IGS type. One representative strain for each IGS type in each sample site (total of 46 strains) was selected for amplification of the 16S rRNA gene using the forward primer P1 (CGGGATCCAGAGTTTGATCCTGGTCAGAACGCT) and reverse primer P6 (CGGGATCCTACGGCTACCTTGTTAC GACTTCACCCC3) [82]. The PCR products were verified as mentioned above, and were sent for commercial sequencing based on the Sanger method (Sangon Biotech (Shanghai, China) Co., Ltd.). The acquired sequences were compared against the NCBI database using the online BLASTN tool, and sequences for type strains of defined *Rhizobium* species sharing similarities greater than 97.0% with the new isolates were extracted. The phylogenetic analysis was conducted in the MEGA 7.0 software [84]. Sequences were aligned using Clustal W and the best model of sequence evolution was selected. Then, the phylogenetic tree was inferred using the maximum likelihood (ML) and the non-parametric bootstrap (500 pseudo-replications) methods. 

DNA fragments of *recA* (coding for DNA recombination protein), *atpD* (encoding for ATP synthase beta chain) and *rpoB* (encoding the RNA polymerase β subunit) were amplified separately by PCR using the primer pairs recA41F/recA640R (TTCGGCAAGGGMTCGRTSATG/ACATSACRCCGATCTTCATGC), atpD255F/atpD782R (GCTSGGCCGCATCMTSAACGTC/GCCGACACTTCMGAACCNGCCTG) and rpoB83F/rpoB1061R (CCTSATCGAGGTTCACAGAAGGC/AGCGTGTTGCGGATATAGGCG), respectively [85,86]. The symbiotic genes *nodC* and *nifH* were amplified using the primer pairs nodC540F/nodC1160R (TGATYGAYATG GARTAYTGGCT/CGYGACARCCARTCGCTRTTG) and nifHF/nifHR (TACGGNAARGGSGGNATCGGCAA/AGCATGTCYTCSAGY TCNTCCA) [87,88]. The PCR conditions were adopted from previous reports [88]. PCR products verification, sequencing and tree construction of each sequenced gene were performed as mentioned above. Furthermore, sequences of *atpD* (297 base pairs), *recA* (289base pairs) and *rpoB* (599 base pairs) genes were concatenated and aligned using Clustal W [89]. Distance calculation, construction of ML trees based on each simple gene and the concatenated housekeeping genes, bootstrap analysis, were performed in MEGA 7.0 as described above. The sequences have been deposited in the NCBI database (accession numbers are indicated on the trees (Figure 1 and Figure 2 and Supplementary Figures S1–S5)). 

Alpha-diversity was calculated to estimate the effective numbers of species (ENS) using three diversity indices (numbers of clusters, exponential Shannon index and the inverse of the Simpson index [90]). 

### 4.5. Correlation Analysis of Soil Properties and Environmental Factors with Rhizobial Communities

The principal component analysis (PCA) was performed using CANOCO version 5.0 [91] to investigate the relationships between soil properties (AN, AP, AK, OM, EC and pH) and environmental factors (Alt, AveTmin, AveTmax and AvePrecp) and the rhizobial community composition based on IGS genotypes and rhizobial species. The distance matrix generated from the response variable (i.e., rhizobial composition data) were based on percentage dissimilarity (i.e., Bray–Curtis dissimilarity) obtained from the 43 IGS genotypes (used to identify rhizobia). This matrix was then correlated to environmental and soil factors.

### 4.6. Symbiotic Efficiency Measurements

Symbiotic efficiency of the representative strains was evaluated on both variety 1 and variety 2 of the local common bean. Briefly, surface-sterilized seedlings were aseptically transferred in pots (1 plant/pot) containing sterile vermiculite as substrate and inoculated with 1 mL of rhizobial suspension (OD_600_ = 1.0). Plants were grown under greenhouse conditions and watered with sterile N-free nutrient solution as required [92]. Symbiotic performance was evaluated 45 days after inoculation for both bean varieties. Common bean growth was estimating by weighting their dry root and shoot biomass, counting the number of nodules and measuring leaf chlorophyll contents (SPAD chlorophyll meter). Uninoculated plants were included as a negative control, and all treatments were performed in three replicates. A symbiotic performance score (%) was calculated for each treatment, considering 25% of the nodulation index (number of nodules per plant), 25% of the leaf chlorophyll index (SPAD value per plant) and 50% of the plant biomass index (dry roots and leaves per plant). Each performance index was standardized by dividing it by the best average value obtained in the experiment (n = 3). Data were analyzed by one-way ANOVA followed by an LSD post hoc test (*p* = 0.001).

### 4.7. Measurements of Abiotic Stress on Rhizobial Strain Growth

Acid and alkali tolerance test: The 46 representative strains of common bean-nodulating rhizobia were inoculated on YMA solid medium at different pH (5, 6, 7, 8, 9, 10 and 11). Each strain was repeated on 3 plates and incubated at 28 °C. Growth results were recorded after 2 and 3 days in comparison with the control at pH 7. 

Salt tolerance test: Representative rhizobial strains were inoculated on YMA solid medium containing different concentrations of NaCl (0.01%, 1%, 2%, 3% and 4% (*w*/*v*)), with 0.01% NaCl plates as control. Each strain was tested in triplicate, and growth was assessed after 2 and 3 days of incubation at 28 °C.

Temperature growth tolerance test: Each representative rhizobium was inoculated in liquid TY medium and cultured at different temperatures (4 °C, 10 °C, 28 °C, 37 °C and 45 °C). Each treatment was replicated 3 times. Optical density (O.D.) was measured at 600 nm after 2 and 3 days of incubation.

Drought tolerance test: Polyethylene glycol 6000 (PEG 6000) was used to artificially simulate drought conditions. Different amounts of PEG were added to YM broth medium, resulting in final concentration of 0%, 3%, 5%, 7%, 10% and 15% (*w*/*v*). Each rhizobium was inoculated into these media (three replicates), and cultures were incubated in a rotary incubator at 28 °C for 2 and 3 days.

Glyphosate tolerance test: Glyphosate was sourced from a commercial product containing 41% (*w*/*v*) glyphosate isopropylamine saline solution in H_2_O, at an effective concentration of 2.16 M. Each rhizobial strain was inoculated on YMA medium supplemented with different concentrations of glyphosate (by adding 0, 0.6 mL/L, 1.2 mL/L and 1.8 mL/L of the 41% glyphosate isopropylamine saline solution). Plates were then incubated at 28 °C for 2 and 3 days.

## Figures and Tables

**Table 1:** Genetic groupings of *Rhizobium* isolates associated with *Phaseolus vulgaris* and their geographical distribution in the different sampling sites.

IGS RFLP Type	Isolate Number	Representative Isolate	MLSA Similarity (%) with ^b^:											Species Identification (Clade in Figure 1)
		WYCCWR No./Origin Site ^a^	*Rso*	*Rac*	*Rec*	*Rhi*	*Rva*	*Rsr*	*Rcr*	*Ran*	*Rph*	*Rch*	*Ret*	
20	9	WYCCWR13672/CDYB-22	99.1	95.8	96.3	94.9	95.1	95.4	95.1	95.1	94.9	94.2	93.4	*Rhizobium sophorae* (C1)
18	9	WYCCWR13777/CNHH-10	95.2	98.6	96.3	95.5	95.3	95	94.9	95	95	95.7	94.3	*Rhizobium acidisoli* (C2)
13	11	WYCCWR14027/DEYH-16	95.9	96.8	99	95.7	95.1	94.6	95	94.9	94.7	95.1	93.7	*Rhizobium ecuadorense* (C3)
10	16	WYCCWR13936/DMDH-21	94.3	94.6	93.9	95.3	94	93.6	93.9	93.6	94.4	95.6	94.8	*Rhizobium* genosp. I (C4)
30	5	WYCCWR14133/BSDB-6	94.2	94.7	94	95.2	94	93.5	93.9	94	94.5	95.6	94.6	
43	1	WYCCWR14114/BSDH-6	94.7	95.3	95.2	97.7	95	95.3	95.7	94.1	96.5	95.2	93.4	*Rhizobium hidalgonense* (C5)
25	6	WYCCWR13998/DWSB-10	94	95.2	95.1	98.5	94.5	94.1	93.9	95.7	94	94.7	93.3	
42	1	WYCCWR13867/DXYH-4	94.5	95.6	95.7	99.8	95.1	94.4	94.3	94.3	94.2	94.9	93.8	
37	3	WYCCWR13972/DWSH-6	94.6	95.7	95.8	99.8	95.2	94.6	94.5	94.5	94.4	95.1	93.6	
41	1	WYCCWR13551/CMDH-27	94.6	95.7	95.8	100	95.2	94.6	94.5	94.5	94.4	95.1	93.6	
8	16	WYCCWR13528/CMDH-4	94.6	95.7	95.8	100	95.2	94.6	94.5	94.5	94.4	95.1	93.6	
32	4	WYCCWR13680/CLFH-5	94.6	95.7	95.8	100	95.2	94.6	94.5	94.5	94.4	95.1	93.6	
39	2	WYCCWR13741/CDHH-14	95.1	95.1	94.6	95	98.9	95.1	96.3	94.4	95.1	95.5	93.4	*Rhizobium vallis* (C6)
12	12	WYCCWR13728/CDHH-1	95.1	95.2	94.9	95	99.3	95.1	96.3	94.3	95.1	95.5	93.4	
28	5	WYCCWR13814/CWDB-3	95.6	95.4	95	95.1	95.3	98.5	96.3	94.9	95.2	95.4	93.5	*Rhizobium sophoriradicis* (C7)
23	8	WYCCWR13632/CDYH-7	95.1	95.4	94.6	93.9	96.2	96.8	98.6	94	95.8	94.8	93.9	*Rhizobium croatiense* (C8)
3	44	WYCCWR14106/BCNB-10	95.1	95.4	94.6	93.9	96.2	96.8	98.6	94	95.8	94.8	93.9	
		WYCCWR13582/CYAH-6	95.1	95.3	94.6	93.9	96.2	96.6	98.4	94	95.8	94.8	93.9	
15	10	WYCCWR13581/CYAH-5	95.1	95.1	94.6	93.8	96.2	96.9	98.6	94	95.9	94.6	93.9	
6	19	WYCCWR13601/CYAH-25	95.2	95.4	94.7	94	96.3	96.7	98.5	94	95.9	94.7	94	
21	9	WYCCWR13663/CDYB-13	95.1	95.3	94.6	93.9	96.2	96.6	98.4	94	95.8	94.8	93.9	
1	153	WYCCWR13630/CDYH-5	95.1	95.8	95.1	95.1	94.9	95.3	95.1	98.6	95.1	95	94.3	*Rhizobium anhuiense* (C9)
		WYCCWR13646/CDYH-21	95.1	95.7	95.1	94.7	94.6	95.1	94.7	98.9	94.7	94.6	94.1	
29	5	WYCCWR13999/DWSB-11	94.2	95.1	94.9	96.3	94.8	94.5	94	98.2	94.5	94.7	93.4	*Rhizobium anhuiense* (C9)
11	13	WYCCWR13604/CYAB-3	94.9	95.3	94.9	94.4	94.5	94.2	94.1	99.7	94.5	94.4	93.7	
34	4	WYCCWR13828/CSBH-8	95	95.3	95	94.5	94.5	94.6	94.3	100	94.6	94.6	93.9	
14	10	WYCCWR13549/CMDH-25	94.6	95.6	95.3	97	95.2	94.6	94.6	95.1	96.3	95.8	94.6	*Rhizobium* genosp. II (C10)
2	63	WYCCWR13564/CMDB-13	94.6	95.1	94.7	94.7	95	95.3	95.8	94.7	98.4	96	94.4	*Rhizobium phaseoli* (C11)
40	2	WYCCWR13810/CNHB-19	94.5	95.1	94.6	94.6	95	95.1	95.8	94.7	98.2	96	94.2	
4	38	WYCCWR13550/CMDH-26	94.5	94.7	94.2	93.9	95	95.5	96.3	94.6	98.9	95.3	94.2	
		WYCCWR13893/DXYB-3	94.5	94.7	94.2	93.9	95	95.5	96.3	94.6	98.9	95.3	94.2	
38	3	WYCCWR13883/DXYH-20	94.5	94.7	94.2	93.9	95	95.5	96.3	94.6	98.9	95.3	94.2	
22	9	WYCCWR14123/BSDH-15	94.7	95.1	94.5	94.1	95.2	95.7	96.6	94.6	99.5	95.4	94.4	
36	3	WYCCWR13915/DXYB-25	94.6	95.1	94.7	94.7	95	95.3	95.8	94.7	98.4	96	94.4	
7	17	WYCCWR13909/DXYB-19	94.6	94.7	94.4	94	95	95.7	96.3	94.6	99.1	95.3	94.4	
16	10	WYCCWR13839/CSBH-19	94.6	95.1	94.7	94.7	95	95.3	95.8	94.7	98.4	96	94.4	
33	4	WYCCWR13823/CSBH-3	94.6	94.7	94.4	94	95	95.7	96.3	94.6	99.1	95.3	94.4	
26	5	WYCCWR13529/CMDH-5	94.6	95.1	94.7	94.7	95	95.3	95.8	94.7	98.4	96	94.4	
9	16	WYCCWR13697/CLFH-22	94.6	95.1	94.7	94.7	95	95.3	95.8	94.7	98.4	96	94.4	
24	6	WYCCWR13654/CDYB-4	93.9	94	94	94.3	95.1	94.2	94.5	94.1	95	96.3	95.1	*Rhizobium* genosp. III (C12)
27	5	WYCCWR14006/DWSB-18	94	95.4	94.6	95.1	95.8	94.3	94.3	94	94.7	98.6	94.7	*Rhizobium chutanense* (C13)
35	3	WYCCWR13808/CNHB-17	94	95.5	94.6	95.1	95.3	94.2	94.2	93.9	95	98.6	94.5	
5	26	WYCCWR13754/CDHB-7	94.5	95.9	95.1	95.1	95.2	94.6	94.6	94.6	95.1	98.6	94.8	
31	4	WYCCWR13563/CMDB-12	93	93.9	93	92.8	93.4	93.1	93.8	92.8	94.3	94.7	97.1	*Rhizobium etli* (C14)
17	9	WYCCWR14090/BLYB-15	93.3	93.3	92.9	92.4	93.4	94.5	93.9	92.3	94.5	94.6	94.2	*Rhizobium* genosp. IV (C15)
19	9	WYCCWR14071/BLYH-17	91.8	91.5	91.7	91.1	91.7	92.3	91.9	91.1	92.9	92.9	92.6	*Rhizobium* genosp. V (C16)
IGS type number: 43	Total isolate number: 608	Total representatives: 46												Total species: 16

a. The collection center number of (WYCCWR)/original isolated number; b. Similarities in MLSA associated with the following type strains-*Rso*: *Rhizobium sophorae*; *Rac*: *Rhizobium acidisoli*; *Rec*: *Rhizobium ecuadorense*; *Rhi*: *Rhizobium hidalgonense*; *Rva*: *Rhizobium vallis*; *Rsr*: *Rhizobium sophoriradicis*; *Rcr*: *Rhizobium croatiense*; *Ran*: *Rhizobium anhuiense*; *Rph*: *Rhizobium phaseoli*; *Rch*: *Rhizobium chutanense*; *Ret*: *Rhizobium etli*.

## Data Availability

The original contributions presented in the study are included in the article/Supplementary Material, further inquiries can be directed to the corresponding author/s.

## Supplementary Materials

The following supporting information can be downloaded at: https://www.mdpi.com/article/10.3390/ijms25158511/s1.
