# Supplementary material for: Unearthing Optimal Symbiotic Rhizobia Partners from the Main Production Area of Phaseolus vulgaris in Yunnan
Source: Int J Mol Sci. 2024 Aug 4;25(15):8511. doi: 10.3390/ijms25158511 (PMC11313401; doi:10.3390/ijms25158511)
Supplement: Supplementary file 1 [file ijms-25-08511-s001.zip › ijms-3049781-supplementary.pdf]

Supplementary Table S1 Soil properties and environmental factors from the different sites studied

Sites$	Physicochemical characteristics of Soils						Environmental factors				
	pH	EC (μS/cm)	OM(g/kg)	AN(mg/kg)	AP(mg/kg)	AK(mg/kg)	Latitude /longitude	Alt(m)	Ave. Tmin°C	Ave. Tmax°C	Ave. Precipitation （mm）per month
CXMD	5.47±0.02 l	117±2.65 e	28.93±0.75 g	126±7.8 efg	77.03±1.5 d	358.33±5.03 a	N101.9167 /E25.4833	2022	10.0	21.5	76.8
CXYA	7.74±0.03 a	245±38.43 c	25.17±0.4 i	146±21.66 bcdef	23.2±2.34 kl	132.67±1.53 g	N101.3833 /E26.0167	2569	6.7	18.3	85.2
CXDY	7.42±0.01 cd	819.33±33.5 a	30.23±0.32 f	151.93±8.01 bcde	26.37±0.6 j	97.57±0.95 j	N101.4667 /E26.0333	2234	8.6	20.4	81.4
CXLF	7.22±0.04 ef	142.67±8.5 de	32.57±0.55 e	167.33±14.55 bc	37.83±0.55 h	132.33±1.53 g	N102.5 /E25.3	1816	10.8	21.9	76.2
CXDH	5.77±0.04 k	74.37±12.36 f	24.8±0.35 i	103.17±3.34 g	22.93±0.4 kl	152.33±0.58 e	N101.4333 /E24.9333	1925	11.0	22.4	78.3
CXWD	7.24±0.03 ef	123.33±10.02 e	36.2±0.17 c	172.47±15.54 b	35.1±0.72 i	200±1.73 d	N101.0833 /E26.0833	2967	4.5	15.9	87.6
CXSB	6.37±0.01 i	80.73±10.71 f	30.73±0.45 f	136.03±4.97 cdef	12.57±0.23 m	108.67±2.08 i	N101.85 /E24.75	1583	13.2	24.5	72.5
CXNH	5.43±0.01 l	76.67±2.53 f	33.97±0.31 d	141.7±6.64 bcdef	21.73±0.42 kl	117.67±0.58 h	N101.5 /E25.1167	1861	11.3	22.8	75.5
DLXY	7.02±0.02 g	170.33±30.86 d	39.2±0.36 b	150.7±14.85 bcde	20.03±0.06 l	212±1.73 c	N100.607 /E25.4649	2013	10.1	21.4	81.6
DLWS	7.35±0.02 d	126±1 e	32.53±0.38 e	159.63±6.21 bcd	46.13±2.2 g	106±1 i	N100.2097 /E25.4025	1770	11.2	22.4	82.1
DLMD	6.93±0.01 h	69.13±6.56 f	29.1±0.26 g	133.47±24.5 defg	93.8±0.56 b	144.67±2.08 f	N100.8714 /E25.4786	1992	10.3	21.7	79.3
DLEY	7.38±0.1 cd	364±25.24 b	62.6±0.87 a	388.87±45.41 a	60.47±0.74 e	143.67±3.51 f	N99.9668 /E26.2324	2106	9.3	20.6	85.9
BSLY	7.16±0.03 f	138.33±9.5 de	19.43±0.31 j	124.5±5.86 efg	80.17±1.76 c	345±2 b	N98.8352 /E25.0591	1047	13.6	25.1	112.7
BSCN	7.52±0.02 b	162.67±15.7 d	17.2±0.52 k	118.1±5.86 fg	49.33±1.5 f	88.7±1 k	N99.447 /E24.8081	1033	14.1	25.8	95.4
BSSD	6.07±0.01 j	120±11.14 e	28±0.7 h	166.87±5.9 bc	138.93±2.25 a	214.67±4.04 c	N99.2394 /E24.571	1864	9.6	21.1	96.8

$. CXSB, Chuxiong Shuang bai, CXWD: Chuxiong Wu ding, CXYA: Chuxiong Yao an, CXMD: Chuxiong Mu ding, CXDH: Chuxiong Dong hua, CXDY: Chuxiong Dayao, CXLF: Chuxiong Lufeng, CXNH: Chuxiong Nanhua, DLEY: Dali Er yuan, DLXY: Dali Xiang yun, DLWS: Dali Wei shan, DLMD: Dali Mi du, BSLY: Baoshan Long yang, BSCN: Baoshan Chang ning, BSSD: Baoshan Shi dian.

Supplementary Table S2 Geographic distribution of *P. vulgaris* nodulating rhizobia at the different sampling sites according to the 43 16S-23S rRNA gene IGS types (each associated with one or two representative strains, total of 46 representatives) and the bean variety used for trapping (H: variety 1 with black seeds/B: variety with white seeds, % of the total isolates (n=608)), and alpha-diversity indices calculated at the Cluster and the IGS levels according to sampling sites.

Species (clade) Representative isolate (WYCCWR no.)	Isolate no/ IGS type	Distribution [number of strains (H/B[a])] of IGS types in the sampling site															Subtotal per clade
		CXMD	CXYA	CXDY	CXLF	CXDH	CXWD	CXSB	CXNH	DLXY	DLWS	DLMD	DLEY	BSLY	BSCN	BSSD	
Rhizobium sophorae **(Clade 1, C1)**																	
CDYB-22 (WYCCWR13672)	9/20	0	0	9(1/8,1.5)	0	0	0	0	0	0	0	0	0	0	0	0	9(1/8,1.5)
Rhizobium acidisoli **(Clade 2, C2)**																	
CNHH-10 (WYCCWR13777)	9/18	1(1/0,0.2)	0	0	0	0	1(0/1,0.2)	0	7(7/0,1.2)	0	0	0	0	0	0	0	9(8/1,1.5)
Rhizobium ecuadorense **(Clade 3, C3)**																	
DEYH-16 (WYCCWR14027)	11/13	0	0	0	0	0	0	3(3/0,0.5)	0	0	0	0	8(4/4,1.3)	0	0	0	11(7/4,1.80)
Rhizobium **genosp. I (Clade 4, C4)**																	
DMDH-21 (WYCCWR13936)	16/10	0	0	0	0	0	0	6(0/6,1)	0	0	1(1/0,0.2)	8(7/1,1.3)	0	0	0	1(0/1,0.2)	21(10/11,3.5)
BSDB-6 (WYCCWR14133)	5/30	0	0	0	0	0	0	0	0	0	0	0	0	0	0	5(2/3,0.8)	
Rhizobium hidalgonense **(Clade 5, C5)**																	
BSDH-6 (WYCCWR14114)	1/43	0	0	0	0	0	0	0	0	0	0	0	0	0	0	1(1/0,0.2)	
DWSB-10 (WYCCWR13998)	6/25	0	0	0	0	0	0	0	0	1(0/1,0.2)	4(2/2,0.7)	1(0/1,0.2)	0	0	0	0	
DXYH-4 (WYCCWR13867)	1/42	0	0	0	0	0	0	0	0	1(1/0,0.2)	0	0	0	0	0	0	
DWSH-6 (WYCCWR13972)	3/37	0	0	0	0	0	0	0	1(1/0,0.2)	0	2(2/0,0.3)	0	0	0	0	0	32(20/12,5.3)
CMDH-27 (WYCCWR13551)	1/41	1(1/0,0.2)	0	0	0	0	0	0	0	0	0	0	0	0	0	0	
CMDH-4 (WYCCWR13528)	16/8	6(6/0,1)	0	0	0	0	3(0/3,0.5)	0	0	2(2/0,0.3)	3(0/3,0.5)	0	2(2/0,0.3)	0	0	0	
CLFH-5 (WYCCWR13680)	4/32	0	0	2(0/2,0.3)	2(2/0,0.3)	0	0	0	0	0	0	0	0	0	0	0	
Rhizobium vallis **(Clade 6, C6)**																	14(12/2,2.3)

CDHH-14 (WYCCWR13741)	2/39	0	0	0	0	2(2/0,0.3)	0	0	0	0	0	0	0	0	0	0	
CDHH-1 (WYCCWR13728)	12/12	0	0	0	0	11(10/1,1.8)	0	0	1(0/1,0.2)	0	0	0	0	0	0	0	
Rhizobium sophoriradicis (Clade 7, C7)																	
CWDB-3 (WYCCWR13814)	5/28	0	0	0	0	0	5(0/5,0.8)	0	0	0	0	0	0	0	0	0	5(0/5,0.80)
Rhizobium croatiense (Clade 8, C8)																	
CDYH-7 (WYCCWR13632)	8/23	0	4(1/3,0.7)	3(3/0,0.5)	1(1/0,0.2)	0	0	0	0	0	0	0	0	0	0	0	
BCNB-10 (WYCCWR14106) 、CYAH-6 (WYCCWR13582)	44/3	0	9(2/7,1.5)	6(6/0,1)	1(1/0,0.2)	0	0	0	0	0	1(0/1,0.2)	0	0	10(4/6,1.6)	10(2/8,1.6)	7(6/1,1.2)	
CYAH-5 (WYCCWR13581)	10/15	0	10(3/7,1.6)	0	0	0	0	0	0	0	0	0	0	0	0	0	90(44/46,14.8)
CYAH-25 (WYCCWR13601)	19/6	0	13(12/1,2.1)	0	0	0	0	0	1(1/0,0.2)	0	0	0	0	5(1/4,0.8)	0	0	
CDYB-13 (WYCCWR13663)	9/21	0	0	3(0/3,0.5)	0	1(0/1,0.2)	0	3(0/3,0.5)	1(1/0,0.2)	1(0/1,0.2)	0	0	0	0	0	0	
Rhizobium anhuiense (Clade 9, C9)																	
CDYH-5 (WYCCWR13630) 、CDYH-21 (WYCCWR13646)	153/1	0	8(7/1,1.3)	24(15/9,3.9)	36(13/23,5.9)	1(1/0,0.2)	0	0	1(1/0,0.2)	6(3/3,1)	17(8/9,2.8)	32(14/18,5.3)	19(7/12,3.1)	2(1/1,0.3)	2(0/2,0.3)	5(4/1,0.8)	
DWSB-11 (WYCCWR13999)	5/29	0	0	0	0	0	0	0	0	0	4(2/2,0.7)	0	1(1/0,0.2)	0	0	0	175(84/91,28.8)
CYAB-3 (WYCCWR13604)	13/11	0	3(0/3,0.5)	0	1(1/0,0.2)	0	0	1(1/0,0.2)	0	2(0/2,0.3)	3(3/0,0.5)	1(0/1,0.2)	0	0	1(0/1,0.2)	1(0/1,0.2)	
CSBH-8 (WYCCWR13828)	4/34	0	0	0	0	0	0	1(1/0,0.2)	0	0	0	0	3(1/2,0.5)	0	0	0	
Rhizobium genosp. II (Clade 10, C10)																	
CMDH-25 (WYCCWR13549)	10/14	5(3/2,0.8)	1(0/1,0.2)	0	0	2(2/0,0.3)	0	0	1(1/0,0.2)	0	0	0	1(0/1,0.2)	0	0	0	10(6/4,1.6)
Rhizobium phaseoli (Clade 11, C11)																	
CMDB-13 (WYCCWR13564)	63/2	19(8/11,3.1)	0	0	2(2/0,0.3)	9(3/6,1.5)	0	13(5/8,2.1)	4(2/2,0.7)	9(7/2,1.5)	0	0	0	4(2/2,0.7)	0	3(1/2,0.5)	170(77/93,28.0)

**CNHB-19 (WYCCWR13810)**	**2/40**	**0**	**0**	**0**	**0**	**0**	**0**	**0**	**2(0/2,0.3)**	**0**	**0**	**0**	**0**	**0**	**0**	**0**	
**CMDH-26 (WYCCWR13550) 、 DXYB-3 (WYCCWR13893)**	**38/4**	**12(2/10,2)**	**0**	**1(0/1,0.2)**	**1(1/0,0.2)**	**3(1/2,0.5)**	**0**	**5(2/3,0.8)**	**7(2/5,1.2)**	**9(3/6,1.5)**	**0**	**0**	**0**	**0**	**0**	**0**	
**DXYH-20 (WYCCWR13883)**	**3/38**	**0**	**0**	**0**	**0**	**0**	**0**	**0**	**0**	**1(1/0,0.2)**	**0**	**1(0/1,0.2)**	**1(0/1,0.2)**	**0**	**0**	**0**	
**BSDH-15 (WYCCWR14123)**	**9/22**	**0**	**0**	**0**	**0**	**0**	**0**	**1(0/1,0.2)**	**0**	**1(0/1,0.2)**	**0**	**2(1/1,0.3)**	**1(0/1,0.2)**	**0**	**0**	**4(4/0,0.7)**	
**DXYB-25 (WYCCWR13915)**	**3/36**	**0**	**0**	**0**	**0**	**0**	**0**	**0**	**1(1/0,0.2)**	**2(0/2,0.3)**	**0**	**0**	**0**	**0**	**0**	**0**	
**DXYB-19 (WYCCWR13909)**	**17/7**	**0**	**0**	**0**	**1(0/1,0.2)**	**0**	**0**	**0**	**1(1/0,0.2)**	**7(4/3,1.2)**	**4(3/1,0.7)**	**4(2/2,0.7)**	**0**	**0**	**0**	**0**	
**CSBH-19 (WYCCWR13839)**	**10/16**	**0**	**0**	**0**	**0**	**2(0/2,0.3)**	**0**	**6(3/3,1)**	**0**	**0**	**0**	**1(1/0,0.2)**	**0**	**1(0/1,0.2)**	**0**	**0**	
**CSBH-3 (WYCCWR13823)**	**4/33**	**0**	**0**	**0**	**0**	**0**	**0**	**4(4/0,0.7)**	**0**	**0**	**0**	**0**	**0**	**0**	**0**	**0**	
**CMDH-5 (WYCCWR13529)**	**5/26**	**4(4/0,0.7)**	**0**	**0**	**0**	**0**	**0**	**0**	**1(0/1,0.2)**	**0**	**0**	**0**	**0**	**0**	**0**	**0**	
**CLFH-22 (WYCCWR13697)**	**16/9**	**0**	**0**	**0**	**2(1/1,0.3)**	**0**	**0**	**0**	**6(0/6,1)**	**4(3/1,0.7)**	**0**	**0**	**0**	**4(3/1,0.7)**	**0**	**0**	
***Rhizobium* genosp. III (Clade 12, C12)**																	
**CDYB-4 (WYCCWR13654)**	**6/24**	**0**	**0**	**2(0/2,0.3)**	**1(0/1,0.2)**	**1(1/0,0.2)**	**0**	**0**	**2(2/0,0.3)**	**0**	**0**	**0**	**0**	**0**	**0**	**0**	**6(3/3,1)**
***Rhizobium chutanense* (Clade 13, C13)**																	
**DWSB-18 (WYCCWR14006)**	**5/27**	**0**	**0**	**0**	**1(1/0,0.2)**	**0**	**0**	**0**	**0**	**0**	**2(0/2,0.3)**	**0**	**2(2/0,0.3)**	**0**	**0**	**0**	
**CNHB-17 (WYCCWR13808)**	**3/35**	**0**	**0**	**0**	**1(1/0,0.2)**	**0**	**0**	**0**	**2(0/2,0.3)**	**0**	**0**	**0**	**0**	**0**	**0**	**0**	**34(16/18,5.6)**
**CDHB-7 (WYCCWR13754)**	**26/5**	**0**	**0**	**0**	**2(2/0,0.3)**	**8(0/8,1.3)**	**0**	**0**	**5(4/1,0.8)**	**3(3/0,0.5)**	**3(0/3,0.5)**	**0**	**4(2/2,0.7)**	**0**	**0**	**1(1/0,0.2)**	
***Rhizobium etli* (Clade 14, C14)**																	
**CMDB-12 (WYCCWR13563)**	**4/31**	**3(1/2,0.5)**	**0**	**0**	**0**	**0**	**0**	**0**	**0**	**1(0/1,0.2)**	**0**	**0**	**0**	**0**	**0**	**0**	**4(1/3,0.7)**
***Rhizobium* genosp. IV (Clade 15, C15)**																	**9(6/3,1.5)**

**BLYB-15 (WYCCWR14090)**	**9/17**	**1(1/0,0.2)**	**0**	**0**	**0**	**0**	**0**	**0**	**0**	**0**	**1(1/0,0.2)**	**0**	**0**	**4(2/2,0.7)**	**3(2/1,0.5)**	**0**	
***Rhizobium* genosp. V (Clade 16, C16)**																	
**BLYH-17 (WYCCWR14071)**	**9/19**	**0**	**1(0/1,0.2)**	**0**	**0**	**0**	**0**	**0**	**0**	**0**	**0**	**0**	**0**	**8(8/0,1.3)**	**0**	**0**	**9(8/1,1.5)**
**Total number of isolates (%)**	**608/43**	**52(27/25, 8.6)**	**49(25/24，8.1)**	**50(25/25，8.2)**	**52(26/26，8.6)**	**40(20/20，6.6)**	**9(0/9，1.5)**	**43(19/24，7.1)**	**44(24/20，7.2)**	**50(27/23，8.2)**	**45(22/23，7.4)**	**50(25/25，8.2)**	**42(19/23，7.0**	**38(21/17，6.3)**	**16(4/12，2.6**	**28(19/9，4.6)**	**/**

[a] · (H/B): H is the black *Phaseolus vulgaris* of variety 1; B is the white *Phaseolus vulgaris* of variety 2.

[b] · DLEY: Dali Er yuan, DLXY: Dali Xiang yun, DLWS: Dali Wei shan, DLMD: Dali Mi du, CXSB, Chuxiong Shuang bai, CXWD: Chuxiong Wu ding, CXYA: Chuxiong Yao an, CXMD: Chuxiong Mu ding, CXDH: Chuxiong Dong hua, CXDY: Chuxiong Dayao, CXLF: Chuxiong Lufeng, CXNH: Chuxiong Nanhua, BSLY: Baoshan Long yang, BSCN: Baoshan Chang ning, BSSD: Baoshan Shi dian.

Supplementary Table S3 Resistance to pH of representative strains

Strains [a]	Acid and alkali resistance (pH)						
	pH=5	pH=6	pH=7	pH=8	pH=9	pH=10	PH=11
BSDH-15 BSDB-6 DEYH-16 BCNB-10	-	-	+	+	-	-	-
CMDH-4 CMDH-25 CMDH-26 CMDH-27 CMDB-12 CMDB-13 CYAH-25 CDHB-7 DWSH-6 DWSB-10 BSDH-6 DWSB-11 DWSB-18	-	-	+	+	+	-	-
CDYH-5 CSBH-3 CYAH-6 CDYB-4 DXYB-19 DMDH-21	-	+	+	+	+	-	-
CMDH-5 CYAB-3 CDYH-21 CDYB-22 CLFH-22 CDHH-1 CSBH-8 DXYH-20 DXYB-3 CSBH-19 CNHB-19 CWDB-3	-	+	+	+	+	+	-
CYAH-5 CDHH-14	-	-	+	+	+	+	+
CDYH-7 CDYB-13 DXYH-4 DXYB-25 BLYH-17 BLYB-15	-	+	+	+	+	+	+

Note:"+" indicates strain growth; "-" no strain growth (three replicates).
[a]: strains in bold are among the most pH-tolerant.

Supplementary Table S4 Resistance to NaCl of representative strains

Strains [a]	NaCl %				
	**0.01%**	**1%**	**2%**	**3%**	**4%**
CMDH-4 CMDH-25 CMDH-27 DMDH-21 CDYB-4 CDYB-22 CLFH-5 CLFH-22 CNHH-10 CNHB-17 CNHB-19 CWDB-3 DXYH-4 BLYB-15 BSDH-6	+	+	-	-	-
CMDH-5 CMDH-26 CMDB-12 CMDB-13 CYAH-5 CYAH-6 CYAB-3 CDYH-5 CDYH-7 CDYH-21 CDYB-13 CDHH-1 CDHH-14 CDHB-7 CSBH-3 CSBH-8 DXYH-20 DXYB-3 DXYB-19 DXYB-25 CSBH-19 DWSH-6 DWSB-10 DWSB-11 DWSB-18 DEYH-16 BCNB-10 BSDH-15 BSDB-6	+	-	-	-	-
BLYH-17 CYAH-25	+	+	+	-	-

Note:"+" indicates strain growth, "-" no strain growth (three replicates).
[a]: strains in bold are among the most NaCl-tolerant.

Supplementary Table S5 Resistance to different temperatures of representative strains

Strains [a]	Temperature°C				
	4	10	28	37	45
CMDH-4 CMDH-5 CMDH-26 CMDH-27 CMDB-12 CMDB-13 CYAH-5 CYAH-6 CYAH-25 CYAB-3 CDYH-5 CDYH-21 CDYB-4 CDYB-22 CLFH-5 CLFH-22 CDHH-1 CDHH-14 CDHB-7 CNHH-10 CNHB-17 CSBH-8 DXYB-19 DXYB-25 CSBH-19 DMDH-21 DWSH-6 DWSB-10 DWSB-18 DEYH-16 BCNB-10 BSDH-6 BSDH-15 BSDB-6	-	-	+	-	-
CMDH-25 CDYB-13 CNHB-19 CWDB-3 CSBH-3 DXYH-4 DXYH-20 DXYB-3 DWSB-11	-	-	+	+	-
CDYH-7 BLYH-17 BLYB-15	-	-	+	+	+

Note:"+" indicates strain growth, "-" no strain growth (three replicates).
[a]: strains in bold are among the most temperature-tolerant.

Supplementary Table S6 Resistance to different polyethylene glycol concentrations of representative strains

Strains [a]	PEG%				
	3%	5%	7%	10%	15%
CMDH-4 CMDH-5 CMDH-26 CMDB-12 CYAH-5 CDYH-7 CLFH-22 CDHB-7 CNHH-10 CNHB-17 CNHB-19 CWDB-3 CSBH-3 CSBH-8 DXYH-4 DXYH-20 CSBH-19 DMDH-21 DWSH-6 CDYH-21	+	+	+	-	-
CMDH-25 CYAH-6 CYAB-3 CDYB-4 CDYB-13 CDYB-22 CDHH-1 CDHH-14 DXYB-25 BLYH-17 BLYB-15	+	+	+	+	-
CMDH-27 CMDB-13 CYAH-25 CDYH-5 CLFH-5 DXYB-3 DXYB-19 DWSB-10 DWSB-11 DWSB-18 DEYH-16 BCNB-10 BSDH-6 BSDH-15 BSDB-6	+	+	-	-	-

Note:"+" indicates strain growth, "-" no strain growth (three replicates).
[a]: strains in bold are among the most PEG-tolerant.

Supplementary Table S7 Resistance to different glyphosate concentrations of representative strains

Strains [a]	Glyphosate mL/L		
	0.6	1.2	1.8
CMDH-4 CMDH-26 CMDB-12 CYAH-5 CYAH-6 CYAH-25 CYAB-3 CDYH-5 CDYH-7 CDYH-21 CDYB-4 CDYB-13 CDYB-22 CLFH-5 CDHH-1 CDHH-14 CDHB-7 CNHB-17 CWDB-3 CSBH-8 DXYH-4 DXYB-3 CSBH-19 DMDH-21 DWSB-11 BLYH-17 BLYB-15	+	+	+
CMDH-25 CMDH-27 CMDB-13 CLFH-22 CNHH-10 DXYB-19 DWSH-6 DWSB-10 DWSB-18 BSDH-15 BSDB-6	+	+	-
CMDH-5 CNHB-19 CSBH-3 DXYH-20 DXYB-25 DEYH-16 BCNB-10 BSDH-6	+	-	-

Note:"+" indicating strain growth; "-" indicates no grow (three replicates).
[a]: strains in bold are among the most glyphosate-tolerant.

Supplementary Table S8 Representative strains with the highest abiotic stress tolerances

Strains	Tolerance to [a]:					
	acidity	alkalinity	NaCl	temperature	PEG	glyphosate
CWDB-3 (*R. sophoriradicis*, IGS28)	pH 6	pH 10	1%	37°C	7%	1.8 mL/L
DXYH-4 (*R. hidalgonense,* IGS42)	pH 6	pH 11	1%	37°C	7%	1.8 mL/L
BLYH-17 (*R.* genosp. V, IGS19*)*	pH 6	pH 11	2%	45°C	10%	1.8 mL/L
BLYB-15 (*R.* genosp. IV, IGS17)	pH 6	pH 11	1%	45°C	10%	1.8 mL/L

[a] based-on data from Tables S3 to S7
